# Using Minimal-Redundant and Maximal-Relevant Whole-Brain Functional Connectivity to Classify Bipolar Disorder

**DOI:** 10.3389/fnins.2020.563368

**Published:** 2020-10-20

**Authors:** Yen-Ling Chen, Pei-Chi Tu, Tzu-Hsuan Huang, Ya-Mei Bai, Tung-Ping Su, Mu-Hong Chen, Yu-Te Wu

**Affiliations:** ^1^Institute of Biophotonics, National Yang-Ming University, Taipei, Taiwan; ^2^Department of Medical Research and Education, Taipei Veterans General Hospital, Taipei, Taiwan; ^3^Department of Psychiatry, Taipei Veterans General Hospital, Taipei, Taiwan; ^4^Division of Psychiatry, Faculty of Medicine, National Yang-Ming University, Taipei, Taiwan; ^5^Institute of Philosophy of Mind and Cognition, National Yang-Ming University, Taipei, Taiwan; ^6^Department of Psychiatry, Cheng-Hsin General Hospital, Taipei, Taiwan; ^7^Brain Research Center, National Yang-Ming University, Taipei, Taiwan

**Keywords:** classification, bipolar disorder, functional connectivity, feature selection, machine learning

## Abstract

**Background:**

A number of mental illness is often re-diagnosed to be bipolar disorder (BD). Furthermore, the prefronto-limbic-striatal regions seem to be associated with the main dysconnectivity of BD. Functional connectivity is potentially an appropriate objective neurobiological marker that can assist with BD diagnosis.

**Methods:**

Health controls (HC; *n* = 173) and patients with BD who had been diagnosed by experienced physicians (*n* = 192) were separated into 10-folds, namely, a ninefold training set and a onefold testing set. The classification involved feature selection of the training set using minimum redundancy/maximum relevance. Support vector machine was used for training. The classification was repeated 10 times until each fold had been used as the testing set.

**Results:**

The mean accuracy of the 10 testing sets was 76.25%, and the area under the curve was 0.840. The selected functional within-network/between-network connectivity was mainly in the subcortical/cerebellar regions and the frontoparietal network. Furthermore, similarity within the BD patients, calculated by the cosine distance between two functional connectivity matrices, was smaller than between groups before feature selection and greater than between groups after the feature selection.

**Limitations:**

The major limitations were that all the BD patients were receiving medication and that no independent dataset was included.

**Conclusion:**

Our approach effectively separates a relatively large group of BD patients from HCs. This was done by selecting functional connectivity, which was more similar within BD patients, and also seems to be related to the neuropathological factors associated with BD.

## Introduction

Bipolar disorder (BD) is an affective disorder characterized by episodic fluctuations in mood. It is one of the leading causes of disability in the world and affects more than 1% of the world’s population (Alonso et al., 2011). Based on the mood episodes that the patients’ experience, BD is categorized into two common subtypes, bipolar I disorder (BDI) and bipolar II disorder (BDII). In BDI, at least one manic episode has to have presented, while in BDII, at least one hypomanic episode and one major depressive episode have to have presented (First et al., 1995). The diagnosis of BD and its subtypes depends on the patient’s subjective symptoms and the presence of observational signs. However, BD is one of the most common mental illnesses to be subject to re-diagnosis, and patients may often have been initially diagnosed as suffering from unipolar depression or schizophrenia (Hirschfeld et al., 2003; Ruggero et al., 2010). Therefore, a search for objective neurobiological markers that can assist with diagnosis is a pressing need, and such a system will then help greatly with future treatment decisions related to BD.

Furthermore, BD is known to be a disease that involves neurobiological deficits (Perry et al., 2018). The activity and connectivity of the brain regions that mediate emotional regulation and reward processing have been found to be disrupted in BD (Chen et al., 2011; Strakowski et al., 2012; Phillips and Swartz, 2014); these include alterations in the activity of various limbic structures, such as the amygdala and hippocampus, as well as prefrontal regions, such as the ventrolateral cortex. Moreover, when a connectivity-based approach has been employed previously to investigate BD, the prefronto-limbic-striatal regions have been found to be the areas associated with the main dysconnectivity in BD (Strakowski et al., 2012; Jiang et al., 2017; Roberts et al., 2017). In a review study by Perry et al. (2018), it was suggested that dysconnectivity is most prominent in the amygdala and prefrontal regions when the reviewed studies are considered; nevertheless, dysconnectivity was also observed in the inferior frontal cortex, medial prefrontal areas, anterior cingulate cortex, thalamus, and several other diverse regions of the cortex. However, these observations were based on group-level inferences and as a result could not be applied directly to the categorization of individual patients. Hence, there is a need to develop an approach that assists individual diagnosis; this was coupled with neuroimaging in order to develop an approach that will be able to distinguish BD patients from healthy controls (HCs) and also distinguish BD patients from patients with other psychiatric disorders.

Unfortunately, the thousands of features present in the data created by neuroimaging lead to the “curse of dimensionality” (Bellman, 1961; Altman and Krzywinski, 2018). As feature dimensionality increases, the statistical results obtained often can be the result of data sparsity, overfitting, or both. The problem is made worse when there is a small sample size; and in this context, in previous studies in this area, the sample sizes have usually been relatively small (Frangou et al., 2017; Rubin-Falcone et al., 2018; Squarcina et al., 2019). Furthermore, proper feature selection strategies and reducing irrelevant/redundant data will be able to improve the classification and prediction performance, enhance the ability to generalize, and provide a better interpretation of the learning process. In addition, previous studies have shown that heterogeneity is present in common psychiatric disorders (Jablensky, 2006; Charney et al., 2017). A reduction in heterogeneity should increase the predictive accuracy when diagnosing psychiatric disorders (Insel et al., 2010; Wu et al., 2017; Dwyer et al., 2018). Therefore, minimum redundancy/maximum relevance (mRMR) was used in the present study during feature selection; this uses mutual information quotient as the value of feature importance, calculated by the mutual information of a feature regarding the response divided by sum of that of other features, in order to select the features that show minimal redundancy and maximal relevance to the category being investigated (Peng et al., 2005). It is one of a number of filter-based feature selection methods that are available and is independent of the model being developing. This means that it is unlikely to result in the model suffering from overfitting and also helps to increase efficiency during computation (Guyon and Elisseeff, 2003).

Therefore, the aim of the present study was to develop and validate a classification approach for BD using a large sample of the patients with BD that included both BDI and BDII patients, in conjunction with a well-matched group of HCs and to do this by using whole-brain functional connectivity analysis. Furthermore, mRMR was utilized as a selection method in order to remove irrelevant and redundant features, to avoid overfitting, and to help interpret the functional connectivity; this would be beneficial when distinguishing BD patients from HCs at the individual level. In addition, support vector machine (SVM) was used as the classifier in the present study; this has been widely used for the classification of psychiatric disorders and has often produced very promising results (Costafreda et al., 2011; Wei et al., 2013; Jie et al., 2015; Rashid et al., 2016). This is because the algorithm shows very good performance when attuning non-linear discriminant functions (Wu et al., 2020).

## Materials and Methods

### Participants

A total of 185 health controls and 222 patients with BD were recruited. The patients included both outpatients and inpatients who attended Taipei Veterans General Hospital, Taiwan. Each patient diagnosis was confirmed by an experienced physician according to the “*Diagnostic and Statistical Manual of Mental Disorders*, Fourth Edition” (First et al., 1995) and was based on structured clinical interviews. Potential participants were excluded if they had a neurological illness or any other disorder that affects cerebral metabolism, had substance abuse history or dependence during the previous 6 months, or had a history that included head injury with a documented sustained loss of consciousness and/or neurological sequelae. The clinical assessment of the patients with BD involved using the young mania rating scale (YMRS) and the Montgomery–Åsberg depression rating scale (MADRS), but only some of the patients received a complete rating. The patients were being treated with a variety of atypical antipsychotics, antidepressants, and mood stabilizers before participating in the study. The investigation was conducted according to the latest version of the Declaration of Helsinki. All participants gave written informed consent prior to their participation, and this was after the procedures had been fully explained to them. The present study was approved by the Research Ethics Committee of Taipei Veterans General Hospital.

### Resting-State Functional and Structural Magnetic Resonance Imaging

Scanning was conducted at the Taipei Veterans General Hospital and was carried out on a 3.0-T GE magnetic resonance imaging (MRI) scanner (GE Healthcare Life Sciences, Little Chalfont, United Kingdom) with a quadrature head coil. The head of each subject was immobilized using a vacuum-beam pad inside the scanner. All participants wore earplugs to muffle outside noise. Resting-state functional images were obtained using a T2^∗^-weighted gradient-echo, echo-planar sequence [repetition time (TR) = 2,500 ms, echo time (TE) = 30 ms, flip angle (FA) = 90°, and voxel size = 3.5 mm × 3.5 mm × 3.5 mm]. A total of 200 MRI volumes of each subject were obtained with their eyes closed. A functional whole-brain image volume consisted of 43 interleaved horizontal slices, all of which were parallel to the intercommissural plane. Furthermore, anatomical whole-brain image volumes were obtained using a sagittal magnetization-prepared rapid acquisition gradient-echo three-dimensional T1-weighted sequence (TR = 2,530 ms, TE = 3 ms, echo spacing = 7.25 ms, FA = 7°, field of view = 256 × 256 mm, voxel size = 1 mm × 1 mm × 1 mm) in order to allow more efficient spatial registration and the localization of brain activity; this allowed better correction for any anatomical differences present that might affect the interpretation during functional analysis.

### Preprocessing for Resting-State Functional MRI

Preprocessing and subsequent analyses of the imaging data were performed using Statistical Parametric Mapping (SPM12, Wellcome Institute of Neurology, University College London, United Kingdom) executed in MATLAB 2019b (MathWorks, Natick, MA, United States). The images were preprocessed based on the following steps: (1) the initial eight volumes were excluded; (2) slice-dependent time shifts were compensated for; (3) head motion was corrected for and participants with a framewise displacement > 0.2 mm were discarded; (4) functional imaging volumes were co-registered with their own anatomical images; (5) spatial normalization into the Montreal Neurological Institute space was performed using a non-linear warping algorithm with resampling at a voxel size of 3 mm × 3 mm × 3 mm; (6) spurious data were regressed out by utilizing the Friston 24-parameter model (Friston et al., 1996), and the data included white matter signals, cerebrospinal fluid signals, and global signals; and (7) band-pass filtering from 0.01 to 0.08 Hz was applied to the imaging data. Subsequently, smoothing was conducted using a 4-mm full-width half-maximum Gaussian kernel. The removal from the study of any participants showing considerable head motion (mean framewise displacement > 0.2) meant that, after the above procedures, there were 192 patients with BD, made up of 103 patients with BDI and 89 patients with BDII, as well as 173 health controls, who proceeded on to the follow-up analysis.

### Feature Extraction, Selection, and Classification

**FIGURE 1:**
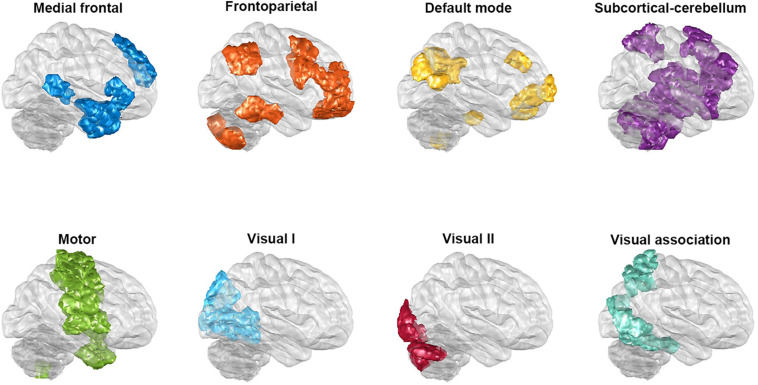
The regions of the eight networks obtained from Shen’s whole-brain functional-connectivity-based atlas. The networks consist of the medial frontal network, the frontoparietal network, the default mode network, the subcortical and cerebellar regions, the motor network, the visual I network, the visual II network, and the visual association network. The glass brains in this figure are shown from a lateral view of the right hemisphere.

Functional connectivity was conducted by parcellating the whole brain into 268 regions, based on Shen’s whole-brain functional-connectivity-based atlas (Shen et al., 2013); this was carried out via a group-wise spectral clustering algorithm. Shen’s atlas categorizes 268 regions into eight networks (see Figure 1); these are the medial frontal network (MFN), the frontoparietal network (FPN), the default mode network (DMN), the subcortical and cerebellar regions (SC), the motor network (MON), the visual I network (VisI), the visual II network (VisII), and the visual association network (VA). The correlation between each of the above pairs of regional time series across the 268 regions was examined using Pearson’s correlation coefficient, and the results were then converted using Fisher’s *r*-to-*z* transformation (Fisher, 1915). Consequently, functional networks for each patient were obtained in the form of 268 × 268 normalized, symmetric correlation matrices. Next, in order to investigate the generalizability of the classification, we used the nested 10-fold cross-validation as the following procedure (also see Supplementary Figure S1). In the outer loop, each sample was separated into 10-folds, and ninefolds were used as the training set, while the remaining fold was used as the testing set. The results of this procedure were used for the classification, and the above process was repeated 10 times until each of the 10-folds had formed testing set. The completed process formed the 10-fold classification used during the present study. During the outer loop of the nested cross-validation, mRMR was used to rank the importance of features with a high correlation with the category but a low redundancy among features. Features before there was a drop in the mRMR feature importance score (the ratio of the mutual information between the feature and the category to that between pairwise features), which represents the feature’s selection confidence, were chosen. Furthermore, to avoid double dipping, feature selection was only applied to the training set. Next, SVM with the Gaussian kernel was utilized and the parameter C and Gaussian kernel scale of the SVM for each training set were determined by 10-fold cross-validation in the inner loop. The trained model and selected features were then applied to the testing set. After all folds of the outer loop had been used as the testing set for classification, the performance in terms of classification, including accuracy, sensitivity, and specificity, was averaged. Given that different parcellations of the 10-folds groupings are likely to influence the performance, the nested cross-validation was run randomly 100 times, and the optimal results identified. In addition, in order to investigate whether the classification procedure outlined above can also be successfully applied to the two common clinical types of BD, namely, BDI (*N* = 103) and BDII (*N* = 89), these two types were separately trained to determine if they are able to be discriminated from the HCs when the sample size is balanced (*N* = 85).

### Inter-Subject Similarity Before and After the Process of Minimum Redundancy/Maximum Relevance

Recently, functional connectivity matrix similarity (alternatively functional connectome fingerprinting) has been developed to allow participant identification to be determined (Finn et al., 2015; Ji et al., 2019; Liu et al., 2019); this is based on the assumption that individuals within the same phenotypic group will have a similar functional connectome. Hence, inter-subject functional connectivity similarity was carried out to investigate the similarity within each group and between BD/HC groups. Specifically, similarity was defined as the cosine distance of every paired functional connectivity matrix in the present study and was calculated before and after the process of mRMR for each complete group (that is not separating the group into training and testing sets). The cosine distance was used as the degree of similarity; and thus the lower the distance, the higher the value, and the higher the similarity.

### Effects of Confounding Factors

There are a number of confounding factors that needed to be taken into consideration during the present study. Firstly, in order to examine the effect of parcellation on the classification performance, a second functional connectivity parcellated using Power’s 264 node-based functional regions of interest (Power et al., 2011) was carried out, and then the same procedures of feature selection and model training were done. Power’s 264-region parcellation was chosen for comparison because it consists of a similar number of regions of interest; if there were fewer nodes used, then this would have resulted in the functional connectivity having a lower resolution. Such a lower resolution might have produced a poorer classification performance, which in turn might have resulted in the functional information essential for discriminating classes fading as the signals from reduced number of parcels became averaged (Arslan et al., 2018). Comparison of the different parcellation was examined using the two-proportion test.

Secondly, in order to rule out the effects of clinical confounding factors, including duration of disease, symptoms, and medication, we investigated the relationship between these factors and the major features repeatedly chosen by mRMR during the outer loop of the nested cross-validation. Pearson’s correlation coefficients were used to examine the continuous variables such as duration and symptom scores. Independent *t*-tests were used to examine categorical variables such as the patient groups with or without atypical antipsychotics, the patient groups with or without antidepressants, and the patient groups with or without mood stabilizers; each of these analyses were carried out separately. In addition, even though head motion had been corrected, spurious functional connectivity may be produced by head motion (Power et al., 2012). Therefore, the difference of head motion estimated via framewise displacement calculation between BD/HC groups, and the correlation of head motion and major features would be evaluated.

## Results

When the demographic data were examined (see Table 1), there were no significant differences in terms of age either between all of the patients and the HCs, or between either of the two clinical subgroups (BDI and BDII) and a subgroup of HCs that had a balanced sample size. However, the sex was not matched in BD/HC and BDII/HC comparisons because there was higher proportion of female in BD and in BDII.

**TABLE 1:** Demographic data.

	Bipolar Disorder	Healthy Control	*p*-Value
Sample size	192	173	
Age	37.16 ± 12.197	35.65 ± 8.934	0.1831
Sex			0.0007*
Male (%)	62 (32.3)	87 (50.3)	
Female (%)	130 (67.7)	86 (49.7)	
Duration	12.45 ± 9.163		
YMRS	3.41 ± 4.429		
MADRS	12.07 ± 10.830		
Medication			
Atypical antipsychotics (%)	97 (50.5)		
Antidepressants (%)	65 (33.9)		
Mood stabilizers (%)	129 (62.5)		
Suicide (%)	47 (24.5)		

	**Bipolar I Disorder**	**Healthy Control**	***p*-Value**

Sample size	103	85	
Age	37.87 ± 11.885	36.53 ± 3.893	0.1831
Sex			0.1011
Male (%)	41 (39.8)	44 (51.8)	
Female (%)	62 (60.2)	41 (48.2)	

	**Bipolar II Disorder**	**Healthy Control**	***p*-Value**

Sample size	89	85	
Age	36.85 ± 11.986	36.53 ± 3.893	0.8123
Sex			0.0001*
Male (%)	21 (23.6)	44 (51.8)	
Female (%)	68 (76.4)	41 (48.2)	

*YMRS, young mania rating scale; MADRS, Montgomery–Åsberg depression rating scale*. *p < 0.05.

### Classification Performance of Bipolar Disorder Versus Healthy Controls With Shen’s 268 Parcellation

**FIGURE 2:**
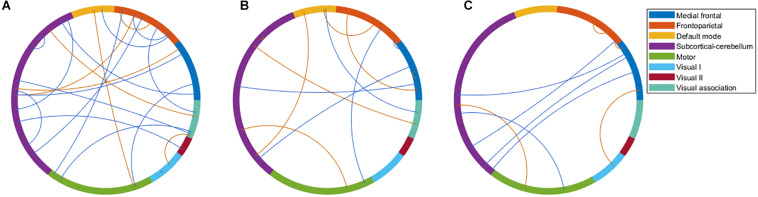
The major features after the 10 times process of minimum redundancy/maximum relevance selection during the outer loop of the nested cross-validation illustrated using the eight networks of Shen’s 268-region parcellation. **(A)** The major features that were selected nine or 10 times during the nested cross-validation of the bipolar disorder and healthy control groups. **(B)** The major features that were selected 10 times during the nested cross-validation of the bipolar I disorder and healthy controls groups. **(C)** The major features that were selected 10 times during the nested cross-validation of the bipolar II disorder and healthy control groups. **(B,C)** were selected 10 times because of the very large number of features selected when nine or 10 times were used. To make the illustration more readable, only the features selected 10 times are shown. Red line represents the connectivity of patients being higher than healthy controls, and blue line represents the connectivity of patients being lower than healthy controls.

**FIGURE 3:**
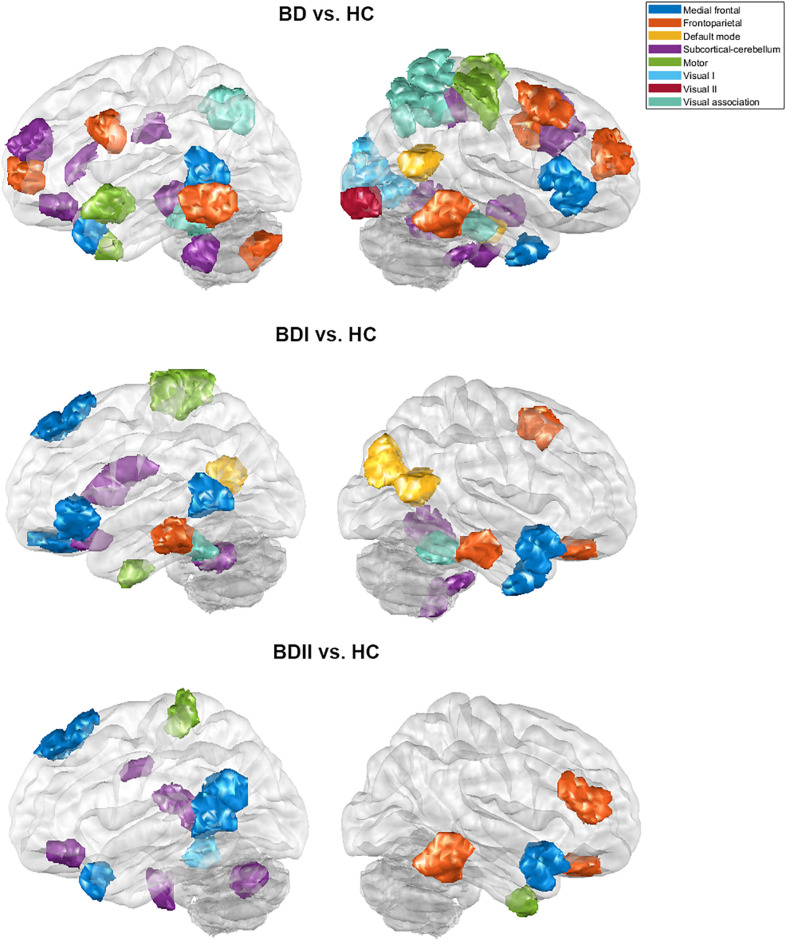
The major features of the classification for the bipolar disorder and healthy control groups, the bipolar I disorder and healthy controls groups, and the bipolar II disorder and healthy controls groups after the 10 times process of minimum redundancy/maximum relevance selection during the outer loop of the nested cross-validation illustrated on the glass brain.

There were 333 features selected on average by the outer loop of all nested cross-validation. The overall accuracies of the training and testing sets based on the features selected using mRMR were 90.69 ± 0.93% and 76.25 ± 1.47%, respectively. For the testing sets, the mean sensitivity and specificity for BD were 77.04 ± 1.64% and 76.71 ± 1.96%, respectively (see Table 2). In addition, the area under the receiver operating characteristic (ROC) curve (AUC) of the classification was 0.840 ± 0.0142. Moreover, there were 22 major features selected by mRMR in a total of nine or 10 times during the outer loop of the optimal nested cross-validation, and these were found to mostly be in the SC, followed by the FPN region (see Figures 2, 3). Since Shen’s parcellation is not restricted by anatomical brain structure, that is, by the gyrus and sulcus, the centroid location of the parcellated region was used to provide more information about the regions. Thus, as can be seen in Table 3, the major features were mainly involved in within and between network connectivity with the SC. The mRMR scores calculated by the whole BD and HC groups were also presented in Table 3. In addition, in order to investigate the importance of the selected features with high mRMR scores, the classification performance was also examined using the same feature numbers of the average selected features mentioned above, but this time with an mRMR low score. The mean accuracies obtained when classifying the testing set under these circumstances were 71.15 ± 1.94%, which was on the trend toward significantly worse performance than the mean accuracy of the testing sets using the features with high mRMR scores (*p* = 0.0594). For the low mRMR score testing sets, the mean sensitivity and specificity were 71.20 ± 2.13% and 72.77 ± 2.38%, respectively, and the mean AUC was 0.772 ± 0.0213.

**TABLE 2:** The classification performance of the testing sets.

Parcellation	Targets	Accuracy (%)	Sensitivity (%)	Specificity (%)	AUC
Shen 268	BD vs. HC	76.25 ± 1.47	77.04 ± 1.64	76.71 ± 1.96	0.840 ± 0.0142
	BDI vs. HC	73.47 ± 1.89	74.87 ± 2.00	73.91 ± 2.71	0.805 ± 0.0165
	BDII vs. HC	72.78 ± 3.18	74.04 ± 3.56	76.24 ± 2.57	0.778 ± 0.0450
Power 264	BD vs. HC	74.36 ± 1.80	75.39 ± 1.76	74.73 ± 2.34	0.818 ± 0.0155
	BDI vs. HC	73.25 ± 2.30	74.88 ± 2.28	73.59 ± 3.04	0.806 ± 0.0200
	BDII vs. HC	70.97 ± 3.31	72.19 ± 3.68	74.69 ± 2.85	0.752 ± 0.0458

**AUC, area under the receiver operating characteristic ROC curve; BD, bipolar disorder; BDI, bipolar I disorder; BDII, bipolar II disorder; HC, healthy control*.*

**TABLE 3:** The major features after the 10 times process of minimum redundancy/maximum relevance selection.

Regions 1 (With Region Label)					Regions 2 (With Region Label)				mRMR Score
**BD vs. HC**									
186	Left superior temporal pole	in	MFN	–	152	Left middle orbitofrontal cortex	in	SC	0.0261
242	Left crus II of cerebellum	in	FPN	–	70	Right inferior temporal cortex	in	FPN	0.0225
188	Left superior temporal pole	in	MON	–	96	Right parahippocampus	in	DMN	0.0191
201	Left inferior temporal cortex	in	VA	–	31	Right precentral cortex	in	FPN	0.0181
131	Right pons	in	SC	–	43	Right angular gyrus	in	VA	0.0170
80	Right calcarine	in	VisI	–	79	Right lingual gyrus	in	VisI	0.0084
199	Left inferior temporal cortex	in	FPN	–	24	Right supplementary motor area	in	MON	0.0054
189	Left middle temporal pole	in	MON	–	57	Right inferior temporal cortex	in	MFN	0.0038
104	Right lobule X of cerebellum	in	SC	–	101	Right lobule IV, V of cerebellum	in	SC	0.0026
198	Left fusiform gyrus	in	VisI	–	41	Right superior parietal cortex	in	VA	0.0023
110	Right lobule VI of cerebellum	in	SC	–	99	Right hippocampus	in	SC	0.0022
157	Left inferior opercular frontal cortex	in	FPN	–	152	Left middle orbitofrontal cortex	in	SC	0.0021
157	Left inferior opercular frontal cortex	in	FPN	–	14	Right middle frontal cortex	in	FPN	0.0020
233	Left parahippocampus	in	SC	–	81	Right inferior occipital cortex	in	VisII	0.0018
28	Right superior medial frontal cortex	in	SC	–	16	Right inferior triangular frontal cortex	in	MFN	0.0015
192	Left middle temporal cortex	in	MFN	–	136	Left rectus	in	SC	0.0014
252	Left crus II of cerebellum	in	SC	–	144	Left middle frontal cortex	in	SC	0.0013
257	Left caudate	in	SC	–	142	Left middle frontal cortex	in	FPN	0.0013
224	Left middle cingulate cortex	in	SC	–	15	Right middle cingulate cortex	in	SC	0.0011
177	Left superior parietal cortex	in	VA	–	91	Right middle cingulate cortex	in	SC	0.0009
59	Right fusiform gyrus	in	VA	–	33	Right precentral cortex	in	MON	0.0008
50	Right middle temporal cortex	in	DMN	–	9	Right middle frontal cortex	in	FPN	0.0007
**BDI vs. HC**									
238	Left lobule VI of cerebellum	in	SC	–	174	Left paracentral lobule	in	MON	0.0995
108	Right lobule IX of cerebellum	in	SC	–	71	Right fusiform gyrus	in	VA	0.0423
104	Right lobule X of cerebellum	in	SC	–	101	Right lobule IV, V of cerebellum	in	SC	0.0361
148	Left superior medial frontal cortex	in	MFN	–	137	Left rectus	in	MFN	0.0361
202	Left inferior temporal cortex	in	MON	–	30	Right superior frontal cortex	in	FPN	0.0361
260	Left caudate	in	SC	–	151	Left inferior orbitofrontal cortex	in	MFN	0.0361
135	Left inferior orbitofrontal cortex	in	SC	–	57	Right inferior temporal cortex	in	MFN	0.0305
193	Left inferior temporal cortex	in	FPN	–	53	Right middle temporal pole	in	MFN	0.0070
201	Left inferior temporal cortex	in	VA	–	49	Right angular gyrus	in	DMN	0.0065
55	Right inferior temporal cortex	in	FPN	–	50	Right middle temporal cortex	in	DMN	0.0050
192	Left middle temporal cortex	in	MFN	–	4	Right superior orbitofrontal cortex	in	FPN	0.0027
258	Left caudate	in	SC	–	222	Left precuneus	in	DMN	0.0013
**BDII vs. HC**									
186	Left superior temporal pole	in	MFN	–	152	Left middle orbitofrontal cortex	in	SC	0.0567
220	Left middle cingulate cortex	in	SC	–	60	Right inferior temporal cortex	in	MFN	0.0490
253	Left crus I of cerebellum	in	SC	–	4	Right superior orbitofrontal cortex	in	FPN	0.0419
268	Left pons	in	SC	–	148	Left superior medial frontal cortex	in	SC	0.0419
70	Right inferior temporal cortex	in	FPN	–	19	Right middle frontal cortex	in	FPN	0.0354
198	Left fusiform gyrus	in	VisI	–	53	Right middle temporal pole	in	MFN	0.0354
229	Left hippocampus	in	SC	–	172	Left postcentral cortex	in	MON	0.0107
192	Left middle temporal cortex	in	MFN	–	4	Right superior orbitofrontal cortex	in	FPN	0.0105
264	Left thalamus	in	SC	–	183	Left superior temporal cortex	in	MFN	0.0097

**DMN, default mode network; FPN, frontoparietal network; MFN, medial frontal network; MON, motor network; SC, subcortical and cerebellar network; VA, visual association network; VisI, visual I network; VisII, visual II network*.*

### Inter-Subject Functional Connectivity Similarity Before and After the Process of Minimum Redundancy/Maximum Relevance

Before the process of mRMR, the inter-subject similarity of the HC group was significantly smaller between groups than within the HC group (0.3793 < 0.4004, *p* < 0.001), but that of the BD group was slightly greater between groups than within the BD group (0.3793 > 0.3789, *p* = 0.8989). However, after the process of mRMR, the inter-subject similarity of both the HC and BD groups was smaller between the groups than within each group (0.3705 < 0.4339, *p* < 0.001; and 0.3705 < 0.3783, *p* = 0.0680, respectively). Furthermore, in order to explore whether the smaller similarity was affected by the feature number, the features were randomly selected with the same number as the features with high mRMR scores. In these circumstances, the mean inter-subject similarity of the HC group was still significantly smaller between groups than within the HC group (0.3891 < 0.4141, *p* < 0.001), but that of the BD group was also slightly greater between groups than within the BD group (0.3891 > 0.3866, *p* = 0.6109).

### Classification Performance of Bipolar I Disorder Versus Healthy Controls and Bipolar II Disorder Versus Healthy Controls Using Shen’s 268 Parcellation

When the classifications of BDI vs. HC and BDII vs. HC were carried out, 342 and 252 features were on average selected during the outer loop of all nested cross-validation, respectively. The mean accuracies of the training/testing sets were 94.52 ± 0.67%/73.47 ± 1.89% and 89.59 ± 4.08%/72.78 ± 3.18%, respectively. The sensitivity/specificity of the testing sets for BDI and BDII was 74.87 ± 2.00%/73.91 ± 2.71% and 74.04 ± 3.56%/76.24 ± 2.57%, respectively (see Table 2). Moreover, the AUCs of the testing sets were 0.805 ± 0.0165 and 0.778 ± 0.0450 for BDI and BDII, respectively. The above might be the result of greater homogeneity because the major features were chosen nine or 10 times during the outer loop, and this was much larger than for the feature selection when all patients were included. Thus, the major features of BDI and BDII were pinpointed as features that were selected in every fold during the outer loop of the optimal nested cross-validation (see Table 3). Moreover, as Figure 2 shows, the major features of BDII existed mostly within and between SC network connectivity, with a few involving network connectivity of FPN and MFN. However, the major features of BDI showed a wider distribution across the networks than BDII. In addition, when the classification of BDI and BDII was being conducted, 139 features were averagely selected during the outer loop. The mean accuracies of the training/testing datasets were 86.23 ± 2.95%/50.91 ± 2.47%, and the sensitivity/specificity was 53.78 ± 2.29%/47.13 ± 3.46%, respectively. Moreover, the mean AUC was 0.501 ± 0.0234.

### Inter-Subject Functional Connectivity Similarity of Bipolar I Disorder and Bipolar II Disorder Before and After the Process of Minimum Redundancy/Maximum Relevance

In addition, the inter-subject similarities of the HC group and BDI/BDII groups before the process of mRMR were both significantly smaller between groups than within the HC group (0.3809 < 0.4056, *p* < 0.001; and 0.3807 < 0.4056, *p* < 0.001, respectively) but were both slightly smaller between groups than within the BDI/BDII groups (0.3809 < 0.3811, *p* = 0.9753; and 0.3807 < 0.3836, *p* = 0.6005, respectively). However, the inter-subject similarities of the BDI group after the process of mRMR and with randomly selected features were still slightly smaller between the groups than within the BDI group (0.3947 < 0.3961, *p* = 0.8445; and 0.4058 < 0.4092, *p* = 0.5282, respectively). The inter-subject similarities of the BDII group after the process of mRMR and with randomly selected features were smaller but not significantly between the groups than within the BDII group (0.4450 < 0.4586, *p* = 0.0533 and 0.4049 < 0.4094, *p* = 0.4507, respectively), with greater trend after mRMR.

### Classification Performance of Bipolar Disorder Versus Healthy Controls Using Power’s 264 Parcellation

To allow the classification of BD and HC, 339 features using Power’s 264 node-based atlas were on average selected during the outer loop of all nested cross-validation. The mean accuracy levels of the classification were 91.95 ± 1.03% for training sets and 74.36 ± 1.80% for testing sets. The sensitivity/specificity for the testing sets was 75.39 ± 1.76% and 74.73 ± 2.34%, respectively (see Table 2). Moreover, the AUC for the testing sets of the nested cross-validation was 0.818 ± 0.0155. Compared with Shen’s parcellation, both the mean accuracies of testing sets using Power’s parcellation were lower, but the difference was not significant (*p* = 0.5552).

For the classification of BDI vs. HC and BDII vs. HC using Power’s 264 node-based atlas, 353 and 225 features were on average selected during the outer loop, respectively. The mean accuracy levels of the training/testing sets were 96.67 ± 0.85%/73.25 ± 2.30% and 89.27 ± 4.63%/70.97 ± 3.31%, respectively. The sensitivity/specificity for the testing sets for BDI and BDII was 74.88 ± 2.28%/73.59 ± 3.04% and 72.19 ± 3.68%/74.69 ± 2.85%, respectively (see Table 2). Moreover, the AUCs of the nested cross-validation were 0.806 ± 0.0200 and 0.752 ± 0.0458 for BDI and BDII, respectively. Compared with those of Shen’s parcellation, both the mean accuracies of testing sets using Power’s parcellation were not significantly different for BDI vs. HC or for BDII vs. HC (*p* = 0.9601 or *p* = 0.7039, respectively).

### Potential Influence of Various Clinical Confounding Factors and Head Motion

After false discovery rate (FDR) correction for multiple comparisons, there was no significant correlation of the major features with either illness duration or symptom scores. Furthermore, there were no significant differences in the major features between the patients who were being treated with atypical antipsychotics, antidepressants, and/or mood stabilizers or were not being treated. In addition, since that there was a significant difference in gender between the BD and HC groups, the classification only for the male BD and male HC groups was also conducted. The mean accuracies were 74.36 ± 2.04%, and the results of two-proportion test indicated no difference between the whole groups and the male groups in overall performance (*p* = 0.5552). These results suggested that there were few clinical confounding factors that were affecting the classification process. Furthermore, the mean (standard deviation) of mean framewise displacement of BD and HC were 0.090 (0.0360) and 0.085 (0.0381), respectively. There was no significant difference (*p* = 0.2303) between BD and HC. In addition, there was no significant correlation between mean framewise displacement and the major features of BD vs. HC or BDI vs. HC after FDR correction. However, only the functional connectivity between right inferior temporal cortex (in FPN) and right middle frontal cortex (in FPN), one of the major features of BDII vs. HC, was significantly associated with mean framewise displacement (*q* < 0.001).

## Discussion

The present study demonstrated that the patients with BD can be successfully discriminated from HCs with a mean testing accuracy of 76.25% and an AUC of 0.840; this analysis involved a relatively large sample size and used a single imager. The process selected the more relevant and less redundant functional connectivity for the classification. In addition, the classification performance of the present study was robust because nested cross-validation was utilized. The findings indicate that the relevant within-network and between-network connectivity of the regions was mainly within the SC, as well as the FPN; and it was these pathways that played the most important roles during the classification that separated BD from HCs. The reasons for being able to satisfactorily discriminate between BD and HC was that the process selected the functional connectivity that was more similar within BD, and that was related to the neuropathological factors associated with BD; this was possible because a whole-brain functional-connectivity-based atlas was involved.

When discriminating BD patients from HCs, previous studies have shown medium to high levels of accuracy when a variety of features were used including voxel-based morphometry (Mwangi et al., 2016), cortical thickness and skewedness (Squarcina et al., 2019), and functional connectivity (Roberts et al., 2017; Wang et al., 2019). Moreover, as a review study (Claude et al., 2020), which added some more recent studies (Squarcina et al., 2019; Wang et al., 2019), demonstrated that more than half of the studies classifying BD and HC used structural MRI, and, furthermore, the number of studies that used functional MRI was greater than the ones using diffusion tensor images. In general, the classification performances of the studies using functional MRI outperformed those of studies using other modalities. The accuracy levels for classification ranged from 57% to 100% among these studies; however, the studies with relatively high accuracy may have obtained these results due to overfitting because of a small sample size. The studies with an accuracy higher than the median accuracy of these studies, which was 68.2%, almost all had a small sample size, namely, one that was less than 100. For example, one reviewed study discriminated between 12 patients with BD and 25 HCs with 100% accuracy using white matter integrity as the features (Besga et al., 2012). Moreover, as the sample sizes became larger, the classification performance levels were reduced (Claude et al., 2020). Notwithstanding the above, the present study, which has a relatively large sample size, has been able to achieve a high accuracy; this accuracy is higher than the median accuracy of the above previous studies as well as being closed to the minimum threshold of clinical relevance (i.e., 80%).

A number of points need to be noted. Firstly, the acceptable classification of BD during the present study is possibly a consequence of deciding to use functional connectivity with higher similarities within BD patients and avoiding using what seems to be more irrelevant similarities between BD patients and HC; the heterogeneous nature of the BD patients may be relevant to this (Charney et al., 2017). Specifically, in the present study, the mRMR process was performed with this purpose in mind. This is supported by the finding that the inter-subject similarity results show that the within-group similarity of BD patients became greater than the between-group similarity after mRMR, and at this point, the patients with BD became more homogenous within the group. Even though both the between-group and within-group differences that are present both before and after mRMR were not significant for the BD group, there was an observable trend. Furthermore, better classification performance was obtained from the features with a high mRMR score when those with a low mRMR score were used, and this is consistent with the hypothesis that an increase in the homogeneity of the psychiatric patients resulted in better predictive model performance (Wu et al., 2017; Dwyer et al., 2018). However, in the present study, rather than clustering the patients into phenotypes based on their neuroimaging features as was done in previous studies, the homogeneity increase was due to mutual similarities in functional connections. Nevertheless, the results that the classification performance between each clinical subtype (i.e., BDI and BDII) and HCs was not better than that between the whole patient group and HCs in the present study may because of heterogeneity within the clinical subtypes. Previously, there have been inconsistences between clinical subtypes and neuroimaging phenotypes (Wu et al., 2017). Phenotypes derived from neurobiological markers of the transdiagnosed study did not match the diagnostic groups (Itahashi et al., 2020). Also, subtypes of patients using unsupervised learning approaches investigated by previous studies were hardly explained by clinical patterns, including symptoms and treatment responses, and usually were compared in clinical patterns with group level or were defined by featured clinical patterns (Costa Dias et al., 2015; Sun et al., 2015). However, the subtypes based on clinical dimensions, such as the history of suicide, may be consistent in neuroimaging and also be clinically explicable (Houenou et al., 2015; Hozer and Houenou, 2016). Moreover, the fact that there is evidence showing that there are few or no differences between clinical subtype (i.e., BDI and BDII) in terms of neurobiological abnormality may explain this inconsistency (Ha et al., 2009; Ambrosi et al., 2016; Hibar et al., 2016; Janiri et al., 2019). This is supported by our results to some extent because in the present study there is poorer classification performance when discriminating BDI from BDII.

Secondly, the classification results of the present study were able to achieve satisfactory performance because the selected features were specific to the regions associated with BD neuropathology. The major features able to discriminate between the BD and HC groups were those frequently selected during feature selection in the cerebellum, the subcortical regions, and some prefrontal regions. These selections are consistent with dysconnectivity during BD, which is known to involve the prefronto-limbic-striatal regions (Strakowski et al., 2012; Jiang et al., 2017; Roberts et al., 2017; Perry et al., 2018). The cerebellum was long regarded as only acting as a motor coordinator, but it also does seem to have a role as a modulator of non-motor functions, including the processing of emotions and cognitions (Stoodley and Schmahmann, 2009, 2010). Furthermore, the involvement of the cerebellum in affective and cognitive function is supported by evidence that the cerebellum interconnects with regions that are involved in reasoning, emotions, motivation, and various drives (Leiner et al., 1986; Middleton and Strick, 1994; Schmahmann, 1996; Kelly and Strick, 2003). In relation to its affective functions, the cerebellum has been implicated in perceiving and recognizing emotional cues, integrating emotional evaluation, and modulating emotional processing (Shakiba, 2014; Adamaszek et al., 2017). Previous studies have also demonstrated affective disorders that are accompanied by cerebellar abnormalities. Some of these findings have indicated that patients with BD show cerebellar microstructural changes (Ambrosi et al., 2016; Zhao et al., 2016), a decreased cerebellum volume (Baldaçara et al., 2011), and alterations in cerebellar activity and cerebro-cerebellar connectivity. These seem to be present even during different mood states such as depression, mania, and euthymia (Johnson et al., 2018; Wang et al., 2018), when psychosis is present (Shinn et al., 2017), and in the absence of medication (He et al., 2018; Luo et al., 2018; Chen et al., 2019). In addition, the subcortical and prefrontal regions also work synchronously during the regulation of emotion processing (Phan et al., 2002; Yamasaki et al., 2002; Strakowski et al., 2012). The subcortical regions covered by the results of the present study included the thalamus, caudate nucleus, and hippocampus region of the cortex. Moreover, the prefrontal regions, which include the orbitofrontal cortex, dorsomedial prefrontal cortex (including superior frontal cortex), and anterior cingulate gyrus (which is covered by region 15 of Shen’s 268 atlas), were also involved; these regions are highly implicated in the modulation of internal emotional stimuli and automatic emotional responses (Phillips et al., 2008). Our findings are consistent with the assumption that BD is an interoceptive disorder (Perry et al., 2018). This is because they are consistent with the findings regarding the regions involving automatic emotion described in a study by Phillips et al. (2008). Previous studies also indicated that disturbances in emotional regulation are accompanied by abnormalities in the subcortical and prefrontal regions, including enlargement of the gray matter volume, disruption of white matter integrity, altered activation, dysconnectivity, and abnormal properties within functional network (Strakowski et al., 2005; Phillips et al., 2008; Chen et al., 2011; Strakowski et al., 2012; Wang et al., 2017; Perry et al., 2018). Furthermore, the result of the present study, when the focal abnormalities in BDI and BDII patients were compared, demonstrated that the former had more distributed abnormalities as major features when classifying the patients and HCs, which is also consistent with previous studies (Ha et al., 2009; Abé et al., 2016).

In addition to feature selection, the better performance of the present study may be a result of using whole-brain functional-based parcellation. When compared with Power’s 264 node-based atlas, Shen’s 268 whole-brain functional-connectivity-based atlas, with an equivalent number of regions of interest, gave better, although not significantly better, classification performance. These results are consisted with Wang et al. (2019) in which the classification by whole-brain parcellation outperformed that node-based regions-of-interest analysis. Moreover, even though Power’s atlas was created as a spatially continuous parcellation, Arslan et al. (2018) found that it had worse agreement with the regions of task activation, Brodmann areas, and myelinated cortical areas than Shen’s atlas. In addition, even though both the present study and Wang et al. (2019) study demonstrated high accuracy, the features extracted from the present study seem to be more reasonable than an approach using anatomical parcellation for functional features extraction. This is because, when compared with anatomical parcellation, functional-connectivity-based parcellation shows much better agreement with the underlying resting-state functional MRI (rs-fMRI) connectivity (Arslan et al., 2018). For example, Deen et al. (2011) conducted cluster analysis to investigate the subdivisions of insula based on rs-fMRI and found distinct patterns of connectivity within subdivisions of the insula (Deen et al., 2011); this was in spite of the fact that the insula forms a single parcel in a standard anatomical brain atlas (Tzourio-Mazoyer et al., 2002).

There are several limitations that affect the present study. The first is that all the patients were being treated with medication; the drugs included atypical antipsychotics, antidepressants, and mood stabilizers. Such long-term treatment with medication can bring about changes that affect the brain. Nevertheless, in the present study, there was no significant correlation between any of the major features and either illness duration or symptom scores. Furthermore, there also were no significant differences in the major features between the patients on different types of medication. The second limitation is that no independent dataset was included in this study. However, it should be noted that the main purpose of the present study was to investigate the generalizability of the classification procedure used here, rather than an attempt to develop an effective model for assisting diagnosis of BD. Thirdly, the amygdala, which plays an essential role in affective disorders, was not identified as one of the major features used for discriminating BD patients from HCs. In the present study, the amygdala was separated into four distinct subregions based on Shen’s 268 parcellation, and these formed four distinctly different regions of interest; this splitting of the amygdala might mean that each of the independent subregions might not have a strong enough impact to be identified during our procedure.

## Conclusion

The present study demonstrates an effective approach for classifying a relatively large group of individuals into BD patients and HCs; this approach was able to achieve the minimum thresholds for clinical relevance. This was done by selecting homogeneous features and using whole-brain functional connectivity. Furthermore, the features chosen by the selection process were clearly related to various the neuropathological factors relevant to BD. Finally, the parcellation approach utilized in this study is congruous with functional performance and the cytoarchitecture of brain. All of the above are possible reasons why the discrimination was successful using our approach.

## Data Availability Statement

The raw data supporting the conclusions of this article will be made available by the authors, without undue reservation.

## Ethics Statement

The studies involving human participants were reviewed and approved by Institutional Review Board, Taipei Veterans General Hospital. The patients/participants provided their written informed consent to participate in this study.

## Author Contributions

Y-LC conceived the study, performed the analysis, and wrote the manuscript. P-CT collected the data, conducted the clinical assessments, and contributed to the interpretation of the results. T-HH contributed to the design of the study. Y-MB, T-PS, and M-HC collected the data and contributed to the interpretation of the results. Y-TW supervised the findings of the study. All authors contributed to the final manuscript.

## Conflict of Interest

The authors declare that the research was conducted in the absence of any commercial or financial relationships that could be construed as a potential conflict of interest.
