# Supplementary material for: Using Minimal-Redundant and Maximal-Relevant Whole-Brain Functional Connectivity to Classify Bipolar Disorder
Source: Front Neurosci. 2020 Oct 20;14:563368. doi: 10.3389/fnins.2020.563368 (PMC7641629; doi:10.3389/fnins.2020.563368)
Supplement: Supplementary file 1 [file Table_1.DOCX]

Supplementary Material

# Supplementary Materials and Methods

The study design and analyses of the present study was illustrated in Figure S1.


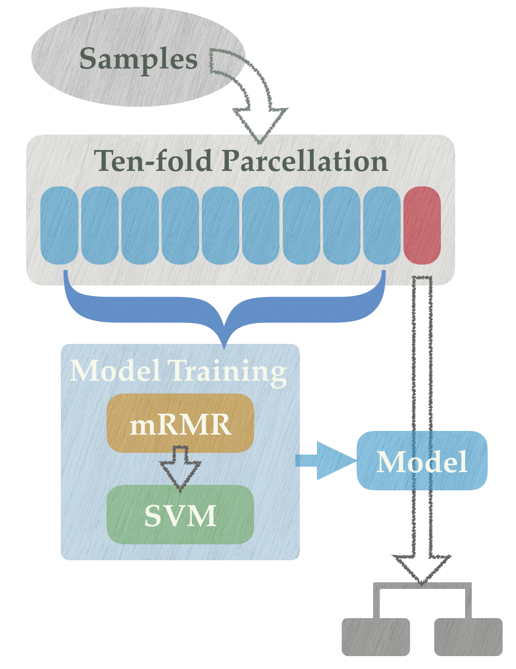


**Supplementary Figure 1.** Flow chart of the present study.

In addition, the classifier utilized in the present study, support vector machine (SVM), is a learning machine for constructing one or several hyperplanes in order to separate the different classes. However, the data are usually linearly non-separable, so they are transformed into a high-dimensional feature space where they can be linearly separable. The hyperplane is described as $\omega^{T}x_{i}+b$, and function$\Phi$ transforms the data into the feature space as $z_{i}=\Phi\left( x_{i} \right)$. The optimal hyperplane in a non-linear space can be attained by minimizing the following formula:

$$\min_{\omega,b,\xi} \frac{1}{2}\omega^{T}\omega+C\sum_{i=1}^{l} \xi_{i}$$

subject to $y_{i}\left( \omega^{T}z_{i}+b \right)\geq1-\xi_{i}$ ,

$\xi_{i}\geq0, i=1,\ldots,l$

where $\xi$ is a slack variable and $C$ is a regularization parameter. The function $\Phi$ can be simplified by using the kernel function, $K\left( x_{i},x_{j} \right)\equiv{\Phi\left( x_{i} \right)}^{T}\Phi\left( x_{j} \right)$. The kernel function used in the present study was the Gaussian kernel:

$K\left( x_{i},x_{j} \right)=e^{\frac{{-\left\| x_{i}-x_{j} \right\|}^{2}}{2\sigma^{2}}}$ .

The kernel scale depends on the adjustable parameter $\sigma$ . Therefore, during the learning process, the penalty parameter $C$ and the kernel scale, more specifically the parameter $\sigma$, are determined for tuning the performance of SVM.
